# Patient experience of telemedicine in addictions

**DOI:** 10.1192/bjo.2021.717

**Published:** 2021-06-18

**Authors:** Soraya Mayet, Iain Mccaw, Zeeshan Hashmani, Zuzana Drozdova, Amelia Gledhill, Samreen Arshad, Shumaila Shahbaz, Thomas Phillips

**Affiliations:** 1 Humber Teaching NHS FT; 2 South West Yorkshire Partnership NHS Foundation Trust; 3 Tees, Esk and Wear Valleys NHS Foundation Trust; 4 Rotherham Doncaster and South Humber NHS Foundation Trust; 5 University of Hull

## Abstract

**Aims:**

Opioid dependence has high risks and opioid substitution treatment (OST) improves outcomes and reduces deaths. Attendance at addiction specialist prescribers may be limited, particularly in rural areas. Telemedicine, such as videoconferencing, can reduce travel and improve access and attendance. Pre-COVID-19, we started a telemedicine service for patients with opioid dependence, prescribed opioid substitution treatment, requiring addiction specialist prescriber consultations. We present patient experience and assess whether patients recommend telemedicine.

**Method:**

Health Research Authority approval for Randomized Controlled Trial of Telemedicine versus Face-to-Face (control) appointments in large semi-rural community addictions service (2500km2) using a modified Hub-and-Spoke (outreach). Adult opioid dependent patients prescribed OST and attending outreach clinics recruited. Participants received two consultations in group. Telemedicine delivered using Skype-for-business videoconferencing. Patients attended outreach clinic, where an outreach worker undertook drug testing and telemedicine conducted via the outreach workers laptop. Specialist addiction prescribers located remotely, at the Hub. Patients self-completed NHS Friends and Family Test (FFT) immediately after appointment, separate from the wider research study. Data collected Sept 2019– March 2020 (pre-COVID-19 lockdown), Microsoft Excel analysis, with qualitative thematic free-text analysis.

**Result:**

Thirty completed FFTs were received, of which all participants were ‘extremely likely’ (n = 19;67%) or ‘likely’ (n = 11;37%) to recommend the Telemedicine service to friends or family, if they needed similar care. Two themes for reasons for recommending the service were; 1. Convenience (reduced travel, reduced travel time and reduced travel costs) and 2. Supportive Staff (including listening, caring and good support). One patient mentioned ‘it is a convenient way to communicate with medical staff, saving time and effort’. Regarding Telemedicine appointments, most participants responded that the timing of telemedicine appointments was good (n = 26;87%), given enough information (n = 30;100%), enough privacy (n = 28;93%), enough time to talk (n = 30;100%), involved as much as they wanted (n = 25;83%), given advice on keeping well (n = 28;93%), and NHS staff were friendly and helpful (n = 29;97%). No participants thought they were treated unfairly. When asked what went well, patient themes were: 1. Everything and 2. Communication (including listening and explaining). One patient stated ‘Everything better, telemedicing good, heard it well, everything improved this year’. In terms of what the service could do better, there were no issues identified.

**Conclusion:**

The Telemedicine in Addictions service was overwhelmingly highly recommended by patients. Patients recommended the service because of convenience and supportive staff. The use of telemedicine is acceptable to patients and could be considered more widely. Due to COVID-19, this technology may be beneficial access to addiction services.

